# *QuickStats*: Percentage*^,^^†^ of Adults Aged ≥20 Years Who Consumed Vegetables on a Given Day, by Race and Hispanic Origin^§^ — United States, 2015–2018

**DOI:** 10.15585/mmwr.mm7027a3

**Published:** 2021-07-09

**Figure:**
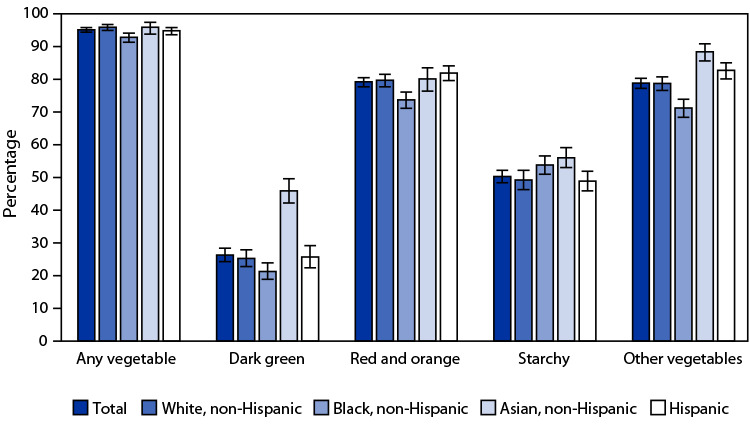
During 2015–2018, 95.1% of adults aged ≥20 years consumed any vegetable, 26.3% consumed dark green vegetables, 79.2% consumed red and orange vegetables, 50.3% consumed starchy vegetables, and 78.8% consumed other vegetables on a given day. Non-Hispanic Black adults were least likely to consume any vegetable (92.8%). Non-Hispanic Black adults were also least likely to consume dark green (21.3%), red and orange (73.7%), and other vegetables (71.2%), and non-Hispanic Asian adults were most likely to consume dark green (45.9%) and other vegetables (88.4%). Non-Hispanic Black (53.8%) and non-Hispanic Asian (56.0%) adults were more likely to consume starchy vegetables.

**Sources:** Ansai N, Wambogo EA. Fruit and vegetable consumption among adults in the United States, 2015–2018. National Center for Health Statistics (NCHS) data brief, no 397. https://www.cdc.gov/nchs/products/databriefs/db397.htm; NCHS, National Health and Nutrition Examination Survey (NHANES) data, NHANES 2017–2018. https://www.cdc.gov/nchs/nhanes.htm

